# The Effects of CuSO_4_ on Membrane Potential and Glutamatergic Synaptic Transmission

**DOI:** 10.3390/biology15151296

**Published:** 2026-08-04

**Authors:** Sara Peters, Katie Neglia, Sonya M. Bierbower, Robin L. Cooper

**Affiliations:** 1 Department of Chemical and Biological Science and Engineering, United States Military Academy, West Point, New York, NY 10996, USA; sara.peters@westpoint.edu (S.P.); sonya.bierbower@westpoint.edu (S.M.B.); 2 Department of Biology, University of Kentucky, Lexington, KY 40506, USA; katie.neglia@uky.edu; 3 The College of Health Sciences, University of Kentucky, Lexington, KY 40536, USA

**Keywords:** copper, glutamate receptor, invertebrates, membrane potential, synapse

## Abstract

Copper sulfate (CuSO_4_) is widely used in industrial and aquatic applications, yet its acute effects on membrane potential and synaptic transmission have not been fully addressed. This study investigated the effects of CuSO_4_ on glutamatergic neuromuscular junctions using larval *Drosophila melanogaster* and Red Swamp Crayfish (*Procambarus clarkii*) as experimental models. Intracellular electrophysiological recordings were used to measure resting membrane potential, evoked excitatory junction potentials (EJPs), and spontaneous miniature EJPs before and during acute CuSO_4_ exposure at multiple concentrations. In larval *Drosophila*, CuSO_4_ produced a rapid, concentration-dependent reduction in both evoked and spontaneous synaptic responses, with higher concentrations nearly abolishing neurotransmission. The co-application of glutamate and CuSO_4_ reduced the sustained depolarization produced by glutamate alone, suggesting antagonistic effects on glutamate receptors. CuSO_4_ also altered the resting membrane potential, producing significant hyperpolarization at lower concentrations. In contrast, crayfish preparations exhibited enhanced synaptic transmission, with increased EJP amplitudes at higher CuSO_4_ concentrations accompanied by membrane hyperpolarization. These findings demonstrate that acute CuSO_4_ exposure differentially affects synaptic transmission and membrane potential across model systems, indicating distinct underlying cellular mechanisms. The results suggest that CuSO_4_ may influence both glutamate receptor function and ion channel activity, providing insight into the neurophysiological effects of copper toxicity and establishing a foundation for future mechanistic investigations.

## Simple Summary

Copper is an essential metal for organisms (animals and plants). It has many uses in biological processes, such as the production of ATP for cellular energy. Copper in the form of CuSO_4_ is also used in industry, as well as in health food supplements. It is an environmental pollutant in high concentrations. The acute effects of copper in high concentrations on cellular processes such as the membrane potential of cells and neuronal communication at synapses are not yet fully understood. This study examines acute exposure of membranes and glutamatergic synapses to CuSO_4_. CuSO_4_ has different effects on crayfish and *Drosophila* neuromuscular junctions and can block some types of glutamate receptors as well as alter membrane excitability. It is important to understand its potential impact on animals, including on humans who consume copper for dietary supplements, in case of over-exposure. Crayfish and *Drosophila* share many similar properties with humans and are used as physiological models.

## 1. Introduction

Copper (II) sulfate (CuSO_4_) is a compound that forms a pentahydrate complex when dissolved in water as CuSO_4_·5H_2_O, which is blue in color. It is used in industrial settings and in ponds to control algal growth from eutrophication and has even been used in reservoirs for drinking water [1]. In the past, CuSO_4_ was used clinically as an emetic [2] and has even been suggested for use by the World Health Organization’s Anatomical Therapeutic Chemical as an emetic. However, it has been found to be too toxic; even 1 g of ingestion can be fatal [3]. It is also toxic for aquatic organisms [4,5,6], and there is concern regarding bioaccumulation within the ecosystem through its transfer from algae to humans [7]. An extensive review on the physiological importance of copper and on its effects on cells, particularly those leading to pathological conditions, is covered by Bulcke et al. [8] and Andrade et al. [9]. In brief, copper can react with oxygen and form superoxide anions, which form radicals [9,10], thus leading to the production of reactive oxygen species (ROS). Copper is known to aggregate with antibodies and potentially lead to plaques with α-synuclein in the brain of vertebrates, leading to neurological disorders [9,11]. The cellular effects of copper toxicity have generally focused on long-term consequences, from several hours to days and years, involving DNA fragmentation and enzymatic dysfunction, such as with the cytochrome P450 oxidative system [9,12,13]. Past studies have directly focused on the toxicity of copper within freshwater benthic invertebrate species (i.e., sludge worm, *Tubifex tubifex*; amphipods *Hyalella azteca* and *Gammarus pulex*, Harlequin fly, *Chironomus riparius*; and blackworm, *Lumbriculus variegatus*) [14] as well as marine invertebrates (i.e., oyster, *Crassostrea virginica*; sand dollar, *Dendraster excentricus*, and the sea urchin, *Strongylocentrotus purpuratus*; and Mediterranean mussel *Mytilus galloprovincialis*) [15].

Since it is well established that various invertebrate and vertebrate species are impacted by the toxicity of copper, it has also been of interest to understand the physiological mechanisms of this toxicity. Bulcke et al. [8] reviewed a number of studies, one of which demonstrated effects on Na^+^/K^+^-ATPase pump from rat brain synaptic plasma membranes [16,17,18] and another on the rabbit kidney [19]. Such actions on the Na^+^/K^+^-ATPase pump would result in cellular dysfunction by altering the membrane potential of all cell types. Blocking of the Na^+^/K^+^-ATPase pump generally has a slow effect, over minutes, on depolarizing the resting membrane potential of cells. However, there has not been comprehensive research investigating the rapid effects, within seconds, of copper on membrane potential and excitatory glutamatergic synaptic transmission in various invertebrate and vertebrate animals. It is important to address not only the potential impact on mammals in relation to human health, but also that of other organisms such as macroinvertebrates, as they help to maintain a vibrant ecosystem within lakes and ponds as well as cleansing water for drinking and restricting the population of mosquitoes [4,20,21].

Copper is an essential metal for all organisms, including humans. It is essential for cytochrome c oxidase in ATP production in the mitochondria and superoxide dismutase (SOD) [22]. It is readily available as a dietary supplement in health food stores and often sold as cupric oxide, cupric sulfate, copper amino acid chelates, and copper gluconate [23]. Overexposure from dietary supplements in humans is not common, but can occur. Treatment entails avoiding any additional exposure, but there is no known process of eliminating it from tissues, except in cases of an acute oral exposure where gastric lavage, chelation or blood transfusion could be performed. Overexposure is known to cause conditions such as Wilson’s disease. There is scant literature on the various effects of acute exposure on tissues such as skeletal muscle, neurons and cardiac cells [24,25,26]. Specifically, the effect of copper on the membrane potential of cells and synaptic transmission at neuromuscular junctions or between neurons is of interest in events of acute overexposure. Copper is found throughout the nervous system due to its physiological roles in cellular function [11,27,28]. It has been reported that copper concentrations with the central nervous system with synaptic clefts in mammals can reach as high as 250 μM [8,29,30]. More research is needed on the potential levels which could occur locally due to neural damage and cellular lysis, which may impact healthy neighboring cells. In the case of an infarct or ischemic stroke, it would be of interest to examine the central nervous system to determine whether the released free Cu^2+^ could contribute to the further growing death-zone known to occur after reperfusion [31,32]; however, there are a number of metals (Zn, Co, Cr, Mo, and Cu) that serve as cofactors for cellular enzymes, which may be a contributing factor when cells lyse. Peripheral cells, such as sensory and motor neurons, skeletal muscle, and cells in other organs (i.e., the liver), may also be suspectable to the effects of copper from localized cellular damage or overexposure. The liver accumulates copper to form ceruloplasmin, which is a primary copper carrier protein for the whole body in mammals [33]. Copper and the binding protein (mCrip2) in skeletal muscle are essential for muscle myoblast and the development of muscle [34]. Therefore, these tissues contain significant levels of copper. However, the acute effects of free copper from damage to such organs or from overexposure on the physiology of a variety of cell types are not yet fully understood.

In this study, two model glutamatergic synaptic junctions were used to assess the effects of acute exposure to Cu(II) (i.e., CuSO_4_). The goal of this study is to draw attention to some unique effects between the model systems and direct effects on membrane potential, as well as on responses to glutamate reception in order to better understand the potential acute effects on the central and peripheral nervous system in many animals (i.e., invertebrates as well as vertebrates). The effects on the resting membrane appear to be independent of the action on glutamate receptors, indicating direct interaction on ion channels such as K2P (K^+^ leak) and NALCN (Na^+^ leak) channels. The advantage of using the larval *Drosophila* and crayfish neuromuscular junctions (NMJs) as synaptic models are that the synaptic responses are graded and generally the muscles do not produce action potentials; thus, the quantal nature of synaptic transmission can be assessed [35]. In addition, spontaneous quantal events are observable as indices to pre- and post-synaptic mechanisms of action by compounds.

## 2. Materials and Methods

### 2.1. Animals

Canton S (CS) *Drosophila melanogaster* flies were used in this study. This strain has been isogenic in the lab for several years and was originally obtained from Bloomington Drosophila Stock Center (BDSC). All animals were maintained in vials partially filled with a cornmeal–agar–dextrose–yeast medium. Red Swamp Crayfish (*Procambarus clarkii*) were obtained from a distribution center in Atlanta, GA, USA, and delivered to a local supermarket in Lexington, KY, USA, from which they were purchased. Throughout the study, midsized crayfish measuring 6 to 10 cm in body length and 12.5 to 30 g in body weight were used. The animals were housed in individual standardized plastic containers with aerated water (20 to 21 °C) and fed dry fish food weekly [36].

### 2.2. Physiology

Early third instar larval *D. melanogaster* were dissected in physiological saline. To monitor the transmembrane potentials of the body wall muscle (m6) of 3rd instar larvae, a sharp intracellular electrode (30 to 40 M resistance) filled with 3M KCl was used to impale the fiber. An Axoclamp 2B (Molecular Devices, Sunnyvale, CA, USA) amplifier and 1 × LU head stage were also used. The preparations were dissected in physiological saline [37,38]: (NaCl 70 mM, KCl 5 mM, MgCl_2_·6H_2_O 10 mM, NaHCO_3_ 10 mM, Trehalose 5 mM, sucrose 115 mM, BES 25 mM, and CaCl_2_·2H_2_O 1 mM, pH 7.1). The physiological saline for the crayfish neuromuscular junctions comprised a modified Van Harreveld’s solution (in mmol/L: 205 NaCl; 5.3 KCl; 13.5CaCl_2_·2H_2_O; 2.45 MgCl_2_·6H_2_O; 10 glucose; 0.5 HEPES adjusted to pH 7.4). The CuSO_4_ was dissolved in the saline. The *Drosophila* saline remained at pH 7.1 with added CuSO_4_; however, the crayfish saline needed to be adjusted with NaOH to a pH of 7.4.

**Figure 1:**
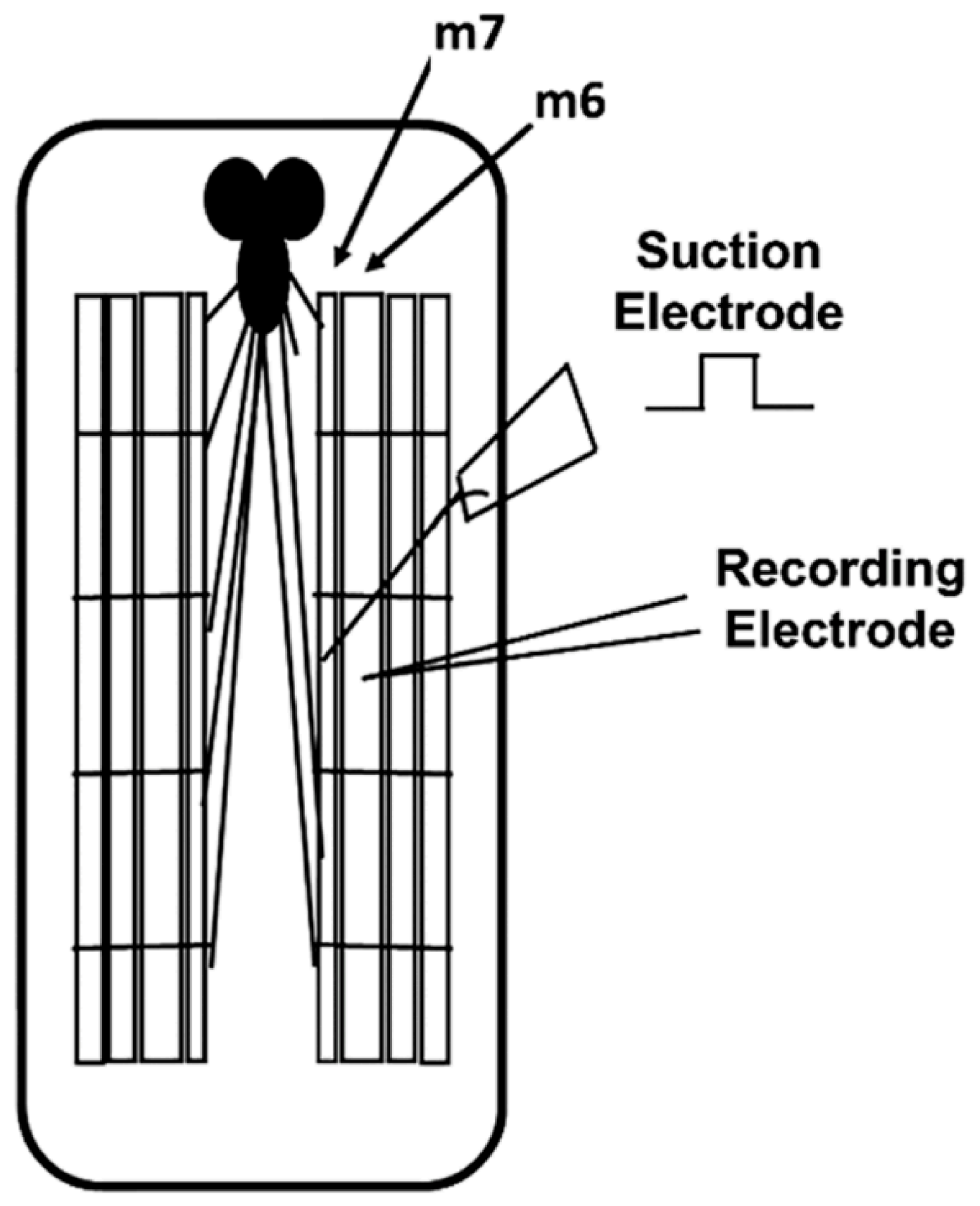
A schematic diagram of the recording arrangement of a 3rd instar larva for obtaining evoked, spontaneous synaptic excitatory junction potentials and resting membrane potential from m6 muscle fiber.

The dissection procedures and electrophysiological measures used for the crayfish are similar to those thoroughly described in text and video by Cooper and Cooper [39] and use similar experimental paradigms as previously described for crayfish and larval *Drosophila* [35,36]. In brief, to obtain synaptic excitatory junction potentials at larval *Drosophila* neuromuscular junctions (NMJs) and resting membrane potentials, the segmental nerves to segment 3 and muscle m6 with segment 3 were used. Third instar larval *D. melanogaster* were dissected along the dorsal midline. The segmental nerves were cut and sucked into a suction electrode, which was filled with saline (the same as detailed above) and stimulated. A glass intracellular electrode (30 to 40 M resistance) filled with 3 M KCl impaled the fiber (Figure 1). An Axoclamp 2B (Molecular Devices, Sunnyvale, CA, USA) amplifier and 1 × LU head stage were used.

**Figure 2:**
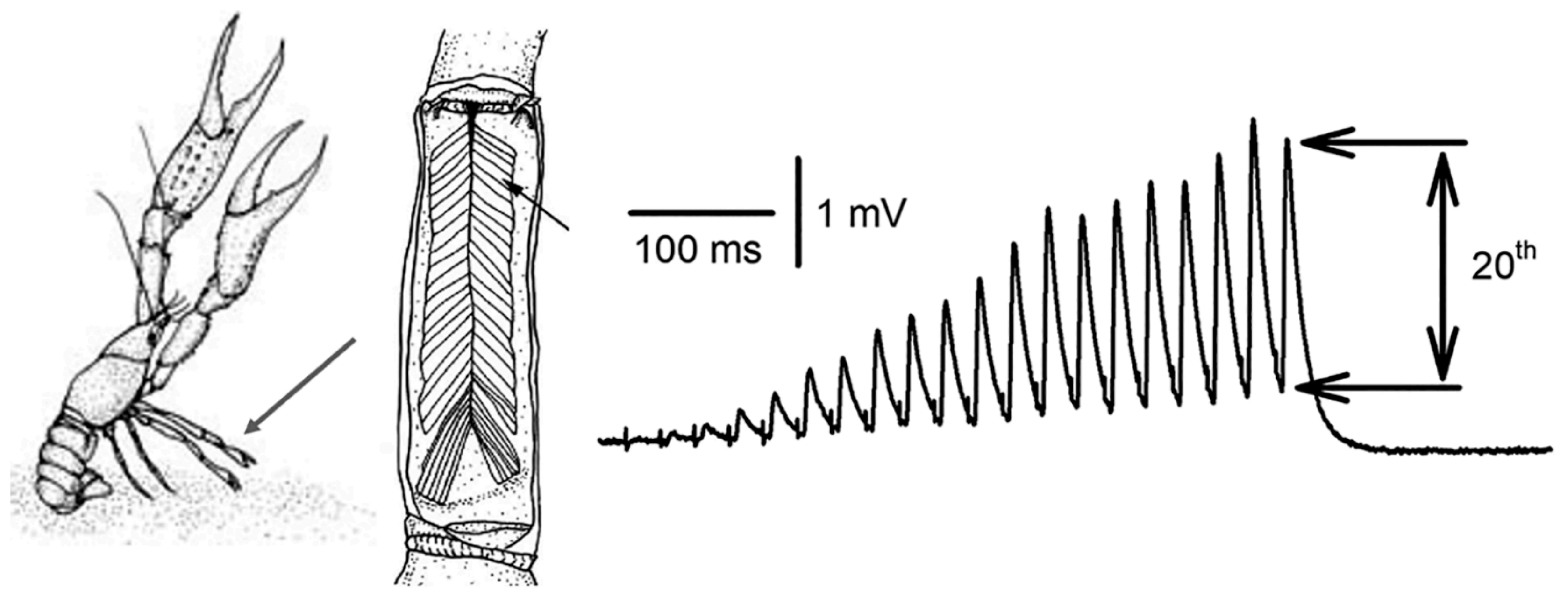
The opener muscle of the crayfish’s first walking leg with facilitated synaptic responses in the excitatory junction potentials from the distal muscle fibers. The synaptic responses depicted show the facilitation which occurs over 20 stimuli. The arrows indicate how the amplitude of the 20th event was measured.

In brief, for crayfish preparation, intracellular recordings of the main distal muscle fibers of the opener muscle fiber in the 1st walking legs were used. The excitatory neuron was isolated from the inhibitory neuron and stimulated in the meropodite segment via pulse trains of 20 stimuli at 40 Hz, separated by 10 s intervals. The amplitude of the 20th EJP was used as an index of synaptic transmission (Figure 2). Responses were recorded with an AxoClamp 2B (Molecular Devices, Sunnyvale, CA, USA) and converted with a PowerLab, 4SP (ADInstruments, Colorado Springs, CO, USA). The recordings for the *Drosophila* and crayfish were obtained and analyzed with Scope or LabChart 7.1 (ADInstruments, Colorado Springs, CO, USA) on a computer with a 20 kHz sampling rate. All experiments were performed at room temperature (20–21 °C).

### 2.3. Statistical Methods

Data from before exposure and during exposure were compared via either a paired *t*-test (for normally distributed data) or a Wilcoxon Signed Rank test (for not normally distributed data). The Shapiro–Wilk test was used for examining normality and the Brown–Forsythe test was used for assessing equal variance.

A *t*-test was used to compare paradigms of varied concentrations. Percentage changes were determined for normalization of the differences among preparations. A significance level of *p* < 0.05 was used in all studies.

## 3. Results

### 3.1. The Effects of CuSO_4_ on Synaptic Responses for Larval Drosophila

**Figure 3:**
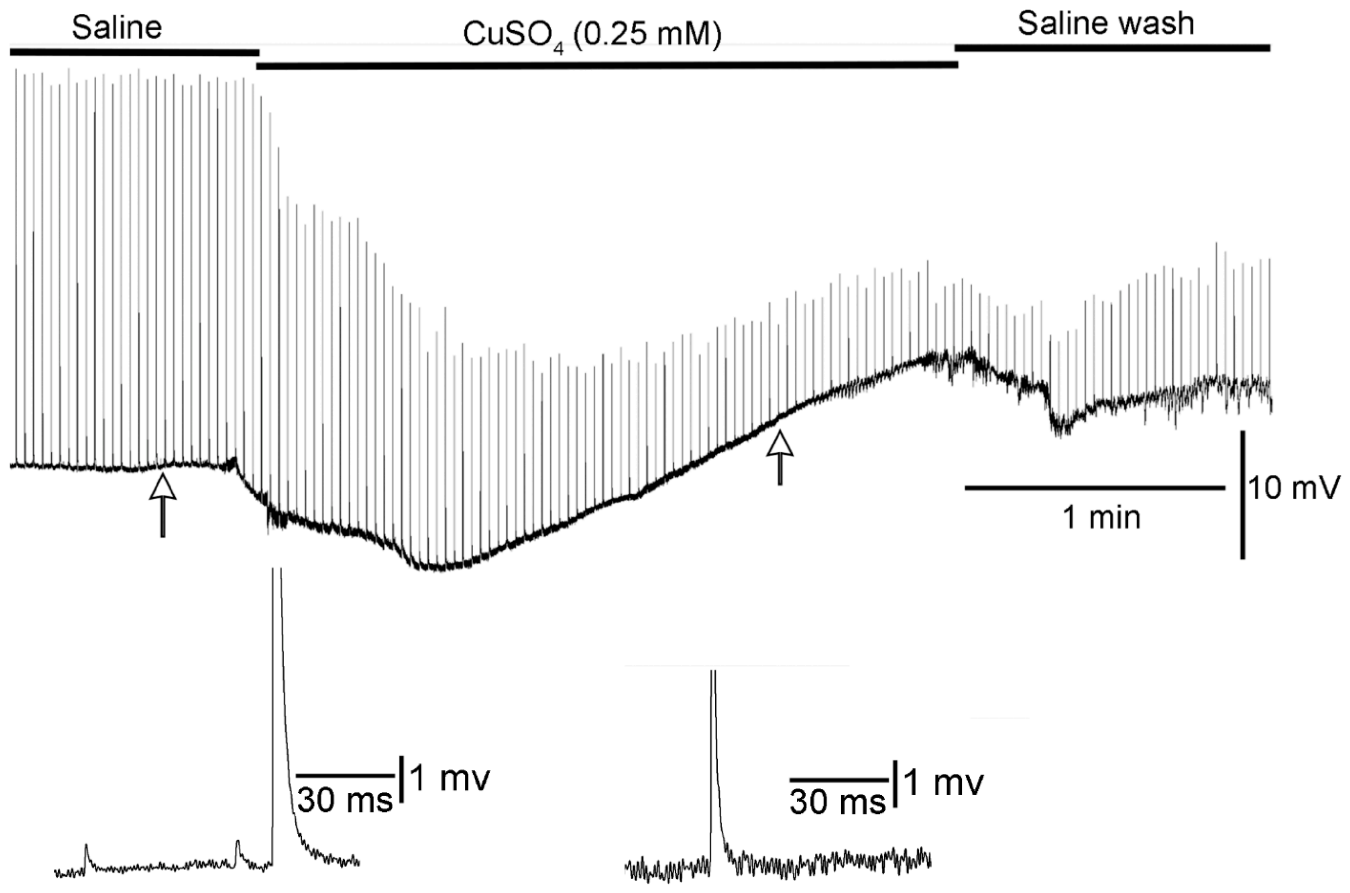
The effects of exposure to low concentration (0.25 mM) of CuSO_4_ on the larval *Drosophila* NMJ. A representative response illustrates the decrease in the EJP amplitude which was evoked every two seconds. Within 2 min, the amplitude was significantly reduced and the spontaneous quantal events decreased in amplitude to become undetectable. The arrows indicate the regions of the trace which were enlarged, shown below the arrow. During the initial saline-only exposure, spontaneous quantal events were observed. Note that the resting membrane potential was hyperpolarized initially with CuSO_4_ exposure and then depolarizes over time.

Evoked EJPs and the spontaneous quantal events (i.e., miniature EJPs) rapidly decreased in amplitude upon exposure to CuSO_4_. The mEJPs became difficult to detect from the baseline noise upon exposure to CuSO_4_ (0.25 mM). After flushing the preparation with fresh saline, and when not containing CuSO_4_, the evoked amplitude started to increase in amplitude, but even after repeated flushing five times, the amplitude did not return to its initial levels. Exposure to CuSO_4_ (0.25 mM) for only 2 min produced more than a 50% decrease in the amplitude of the evoked EJPs. A representative trace in the amplitude of the evoked EJP, with a stimulus delivered every two seconds, illustrates the rapid decline and slight recovery when exchanging the media with fresh saline (Figure 3). This same trend was observed in six of six preparations with a percentage decrease within 2 min by −60.9% +/− 7 (mean +/− SEM) (*p* < 0.05; paired *t*-test comparing within each preparation, n = 6). Thus, it was not practical to determine whether the frequency in the spontaneous quantal events was due to it being undetectable from the baseline.

**Figure 4:**
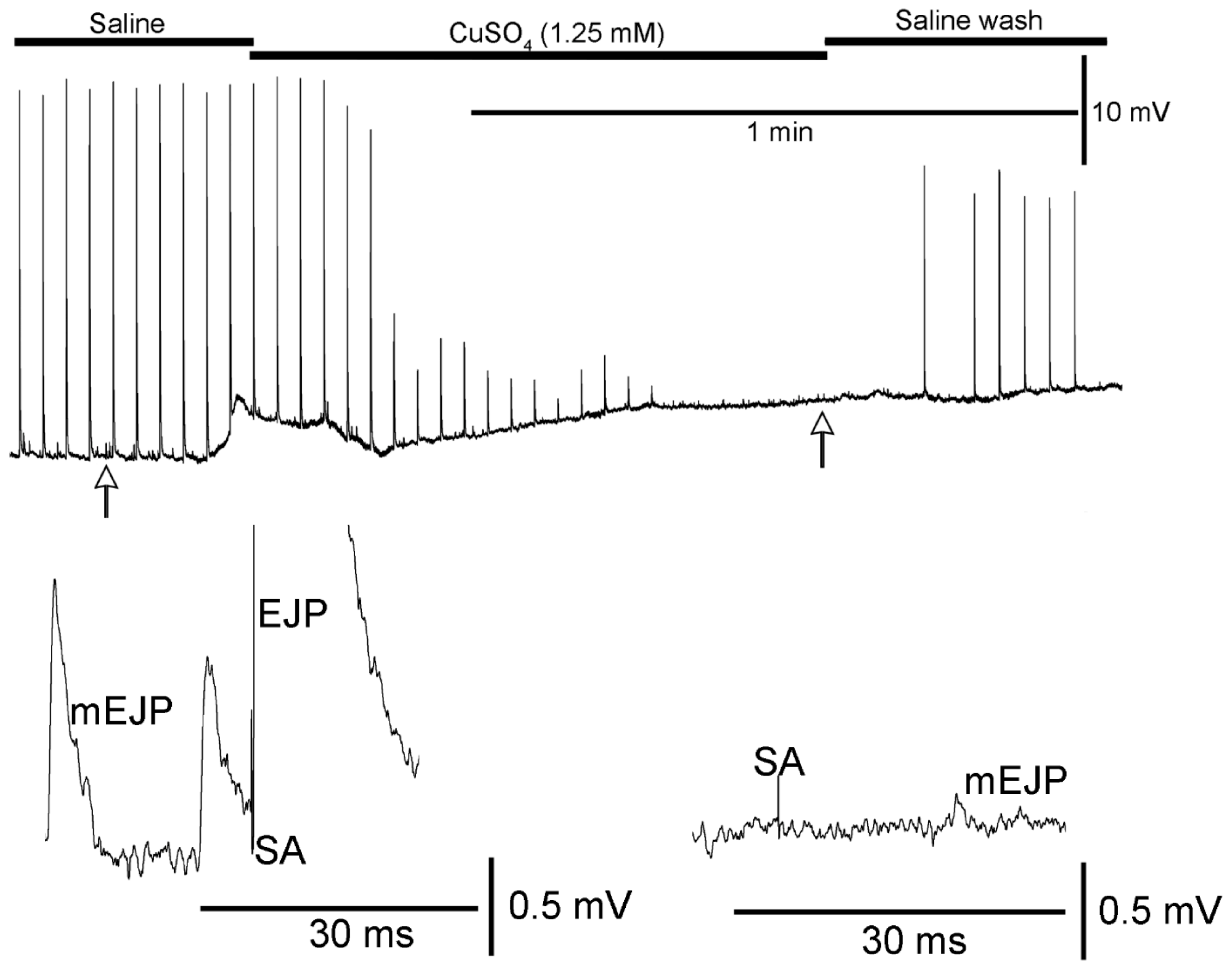
The effects of exposure to high concentration (1.25 mM) of CuSO_4_ on the larval *Drosophila* NMJ. A representative response illustrates the rapid decrease in the EJP amplitude which was evoked every two seconds. Within less than one minute, the amplitude was significantly reduced enough to not be detected after the stimulus artifacts. The spontaneous quantal events of miniature EJPs (mEJPs) also rapidly decreased in amplitude. In time, they were not detectable either. The arrows indicate the regions of the trace which were enlarged, and shown below the arrows. During the initial saline-only exposure, spontaneous quantal events were observed; however, later in time, only stimulus artifacts (SA) without subsequent EJPs occurred during exposure to CuSO_4_. Note that the resting membrane potential did not show hyperpolarization with CuSO_4_ exposure at 1.25 mM; however, the membrane depolarized over time.

The higher concentration of CuSO_4_ (1.25 mM) reduced the amplitude of the EJPs to a level that was not even detectable. The mEJPs also decreased in amplitude quickly (Figure 4). The stimulus artifacts (SA) were detectable when enlarging the trace, with a lack of EJPs during exposure to CuSO_4_. When the bathing media was quickly exchanged, the EJPs returned in some cases, but they did not recover to the full extent. The decrease in EJP amplitude was barely detectable within a minute for each of the six preparations exposed to 1.25 mM (Wilcoxon Signed Rank test, *p* < 0.05, n = 6).

**Figure 5:**
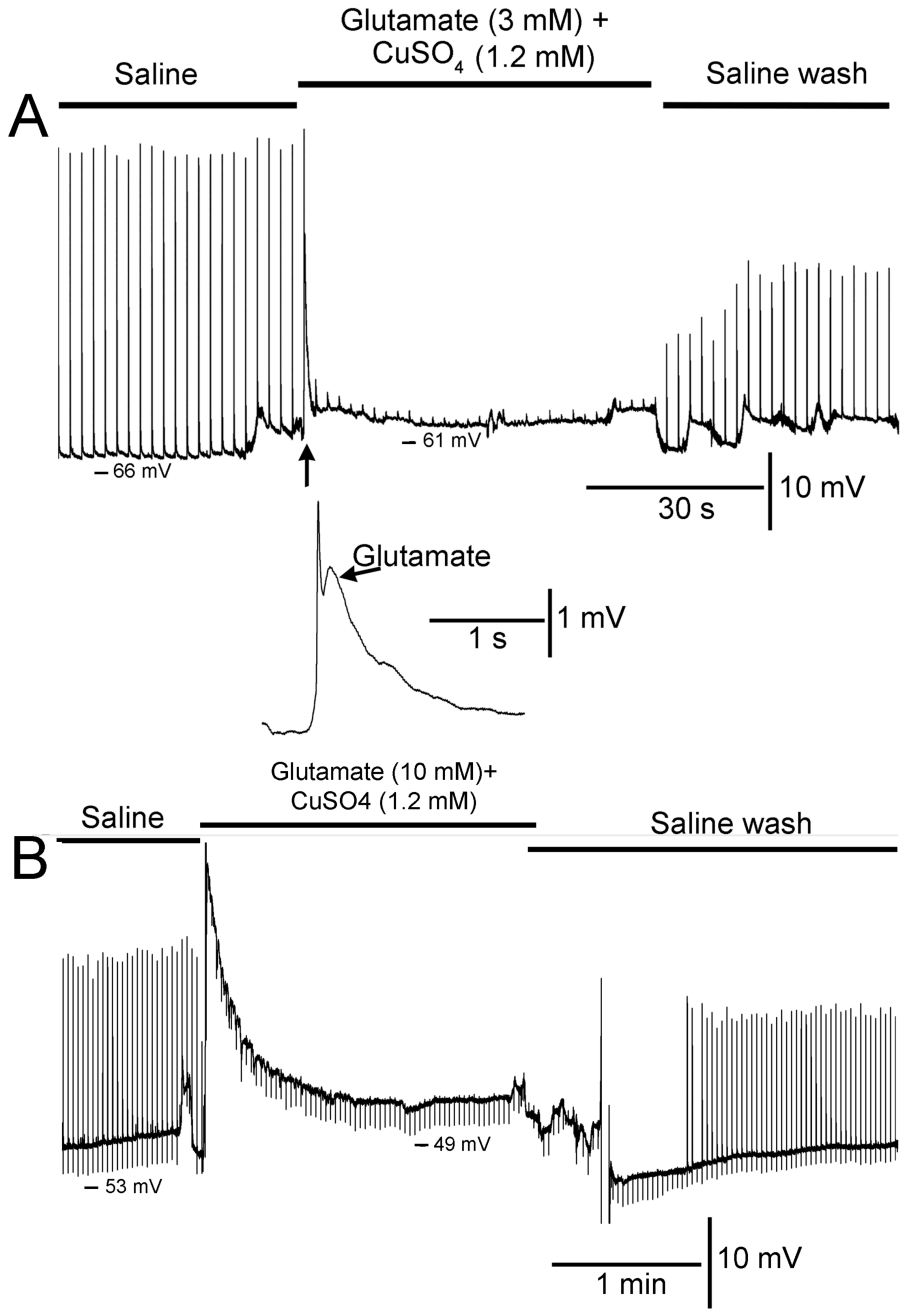
The effect of glutamate combined with CuSO_4_ on membrane potential and synaptic responses. (**A**) Representative response to glutamate (3 mM) combined with CuSO_4_ (1.2 mM). The enlarged inset shows the trace illustrating the depolarization by the exogenous glutamate application at the point of the arrow in the traces. (**B**) Representative response to glutamate (10 mM) combined with CuSO_4_ (1.2 mM). When the compounds are added together, the effect of glutamate produces a rapid depolarization with quick depression of any subsequent evoked responses with a small, sustained depolarization. Evoked stimuli were delivered every two seconds throughout the recording. The stimulus artifacts are present, while the evoked responses are desensitized.

**Figure 6:**
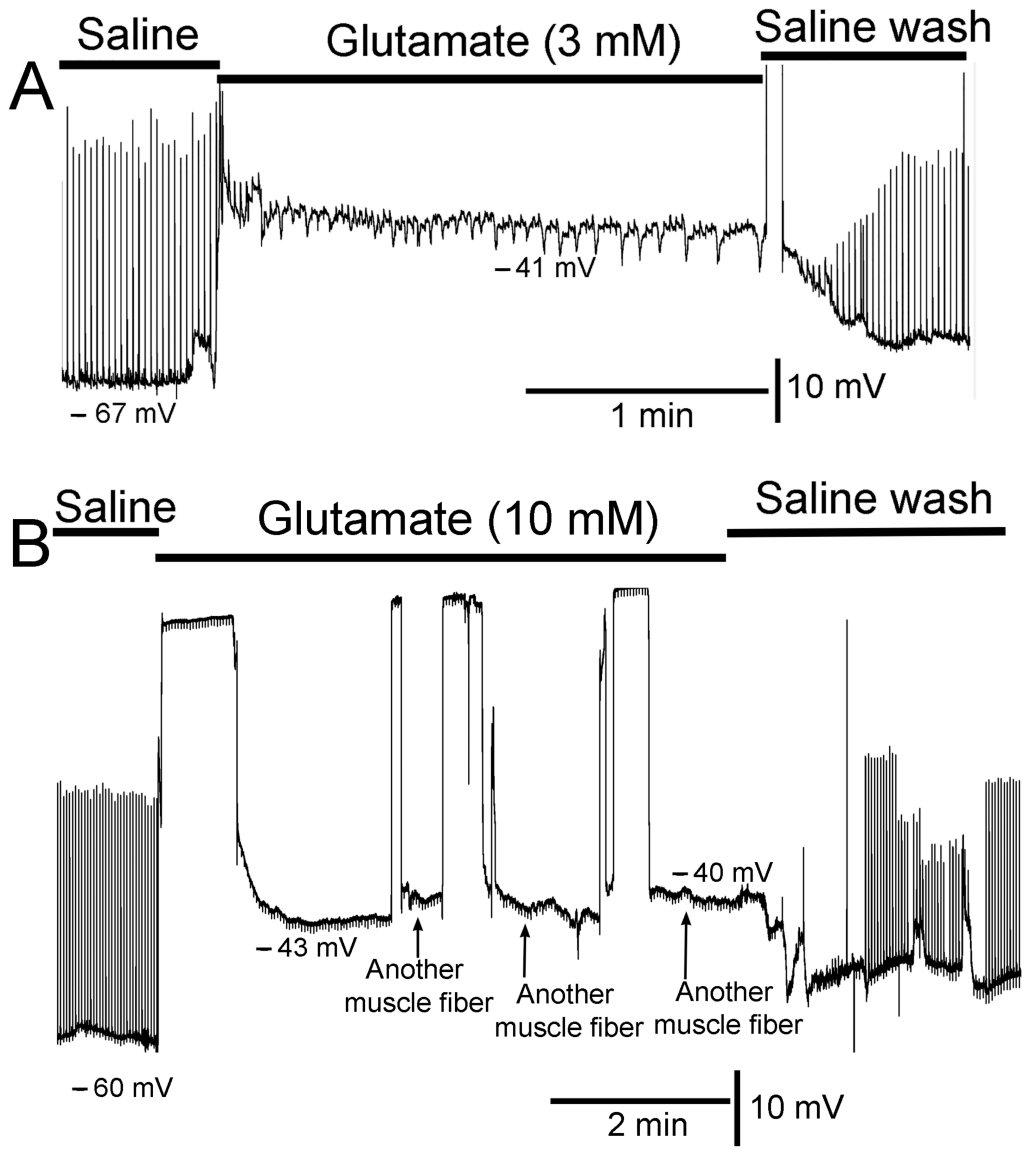
The effect of glutamate alone on membrane potential. (**A**) Application of glutamate (3 mM) rapidly depolarizes the membrane potential and desensitizes synaptic glutamate receptors, reducing evoked EJPs. (**B**) Application of glutamate (10 mM) rapidly depolarizes the membrane potential and desensitizes synaptic glutamate receptors, reducing evoked EJPs. Three other m6 muscle fibers were recorded in the same preparation during the glutamate application. The recording in the first muscle was lost when the electrode was dislodged from the contracting fiber. The next three fibers were still in a slightly contracted state when monitored, but all four fibers had similar membrane potentials while exposed to glutamate (10 mM) and desensitized to evoked glutamate. The membrane potential remains depolarized while exposed to the 3 mM as well as the 10 mM glutamate in the bathing media. Evoked stimuli were delivered every two seconds throughout the recording. The stimulus artifacts are present while the evoked responses are desensitized.

Given that the evoked EJPs and the spontaneous mEJPs decreased in amplitude with exposure to CuSO_4_ in a dose- and time-dependent manner (i.e., more rapid and extreme with 1.25 mM as compared to 0.25 mM), it would appear that CuSO_4_ may be blocking the glutamate receptors, thus decreasing the responsiveness to the glutamate released from the evoked and spontaneous quantal occurrences. To examine this possibility, a combination of glutamate (3 mM or 10 mM) and CuSO_4_ (1.25 mM) was applied while evoking EJPs and monitoring the resting membrane potential. As expected, the evoked EJPs rapidly decreased due to the glutamate receptors’ desensitization, as well as the possibility of CuSO_4_ blocking the glutamate receptors (Figure 5). Interestingly, there was a rapid initial depolarization from the glutamate application, but the sustained depolarization which occurs with continuous glutamate exposure was reduced when co-applied with CuSO_4_ (Figure 5 and Figure 6). To examine whether the contraction with the electrode still in the fiber damaged the membrane and potentially altered the measures of the membrane potential, other fibers were recorded in the same preparation after the initial contractions were over. Similar values for the membrane potential were obtained, indicating a valid measure in the initial fiber monitored (Figure 6B).

**Figure 7:**
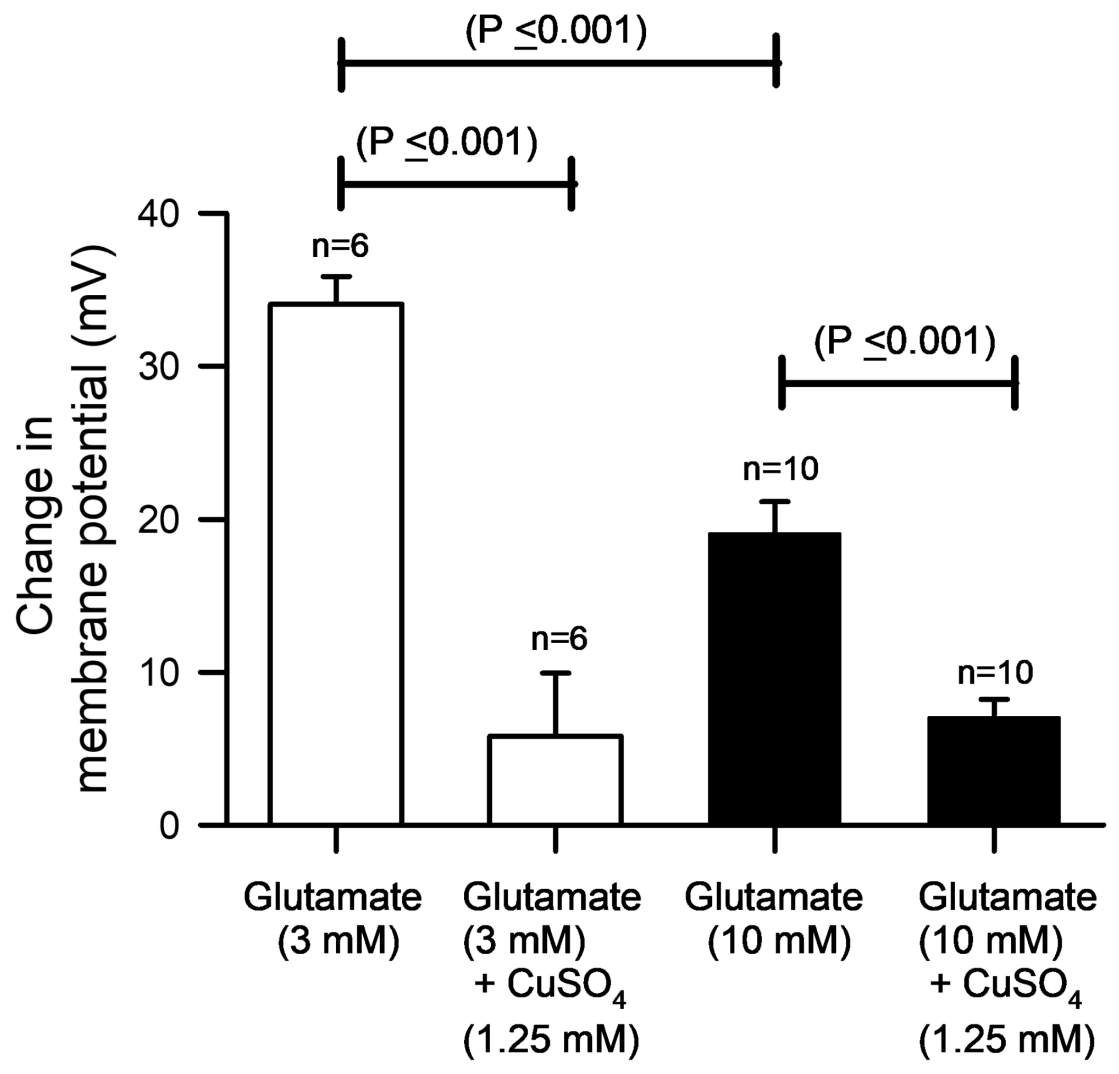
The effect of glutamate on resting membrane potential in the presence of CuSO4. Glutamate alone depolarized the membrane potential and maintained depolarization while glutamate combined with CuSO_4_ also maintained depolarization but not as notably as glutamate alone (*t*-test, *p* < 0.001, n = 6 for each of the 3 mM glutamate exposures and n = 10 for each of the 10 mM glutamate exposures).

The amount of depolarization with exposure to glutamate alone was maintained to a greater extent than glutamate combined with CuSO_4_ (Figure 7: *t*-test, *p* < 0.001, n = 6 for each of the 3 mM glutamate exposures and n = 10 for each of the 10 mM glutamate exposures). The exposure of glutamate at 10 mM did not maintain a consistent depolarization rate to as great an extent as 3 mM glutamate (Figure 7). The 10 mM glutamate dose appeared to relax the muscle fiber sooner after the initial strong contractions. This might have been due to a more rapid desensitization of the receptors.

### 3.2. The Effects of CuSO_4_ on Synaptic Responses for Crayfish

**Figure 8:**
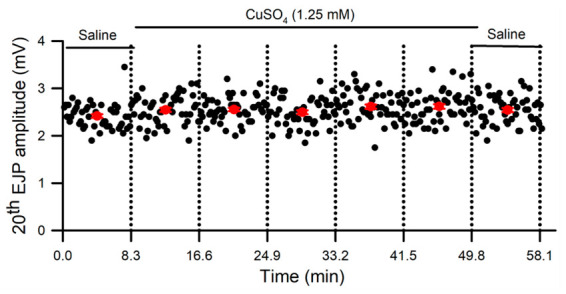
The effects of exposure to 1.25 mM CuSO_4_ on the amplitude of the 20th EJP within the stimulus train. A representative preparation in the 20th EJP amplitude over time with the initial saline, exposure to CuSO_4,_ and exchanging the media for fresh saline without CuSO_4_. The red symbols illustrate the mean (+/−SEM) within the 50 trials.

The exposure to 1.25 mM CuSO_4_ only produced slight increases in the amplitude of the 20th EJP over the exposure time of 50 min (Figure 8). Using the mean values of the 50 responses for each preparation and comparing the time to the initial saline over the period of exposure to CuSO_4_ did prove to be significant (Paired *t*-test, *p* < 0.05, n = 6). However, the average percentage increase for all six preparations was small (13.9 +/− 3.3; n = 6).

**Figure 9:**
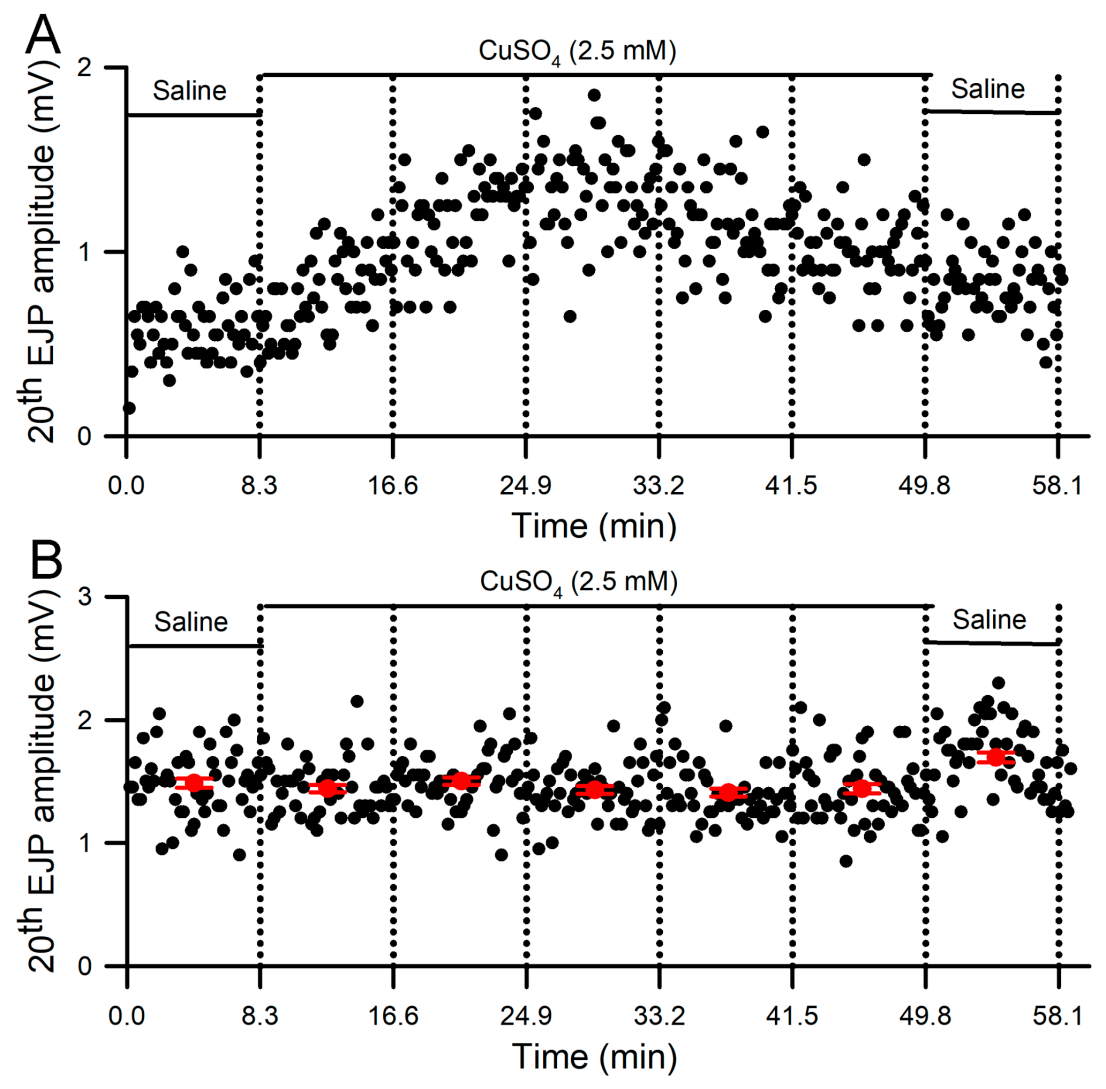
The effects of exposure to 2.5 mM CuSO_4_ on the amplitude of the 20th EJP within the stimulus train. (**A**) Representative preparation illustrating the values in the 20th EJP amplitude over time with the initial saline, exposure to CuSO_4,_ and exchanging the media for fresh saline without CuSO_4_. (**B**) Only one of the six preparations presented a slight increase in the average amplitude of the EJPs in the mean (+/−SEM) of 50 responses after 20 min, while the other preparations showed larger increases. The red symbols illustrate the mean (+/−SEM) within the 50 trials.

With exposure to 2.5 mM CuSO_4_, five of the six preparations showed a substantial increase in the amplitude of the 20th EJP over the exposure time of 50 min. Preparations varied, with some increasing quickly upon exposure while others took 20 min to show a significant increase, as shown (Figure 9A). The one preparation which only showed a slight average increase after 15 min in amplitude is illustrated in Figure 9B. There was an increase in the mean values of the 50 responses for each preparation (paired *t*-test, *p* < 0.05, n = 6).

**Figure 10:**
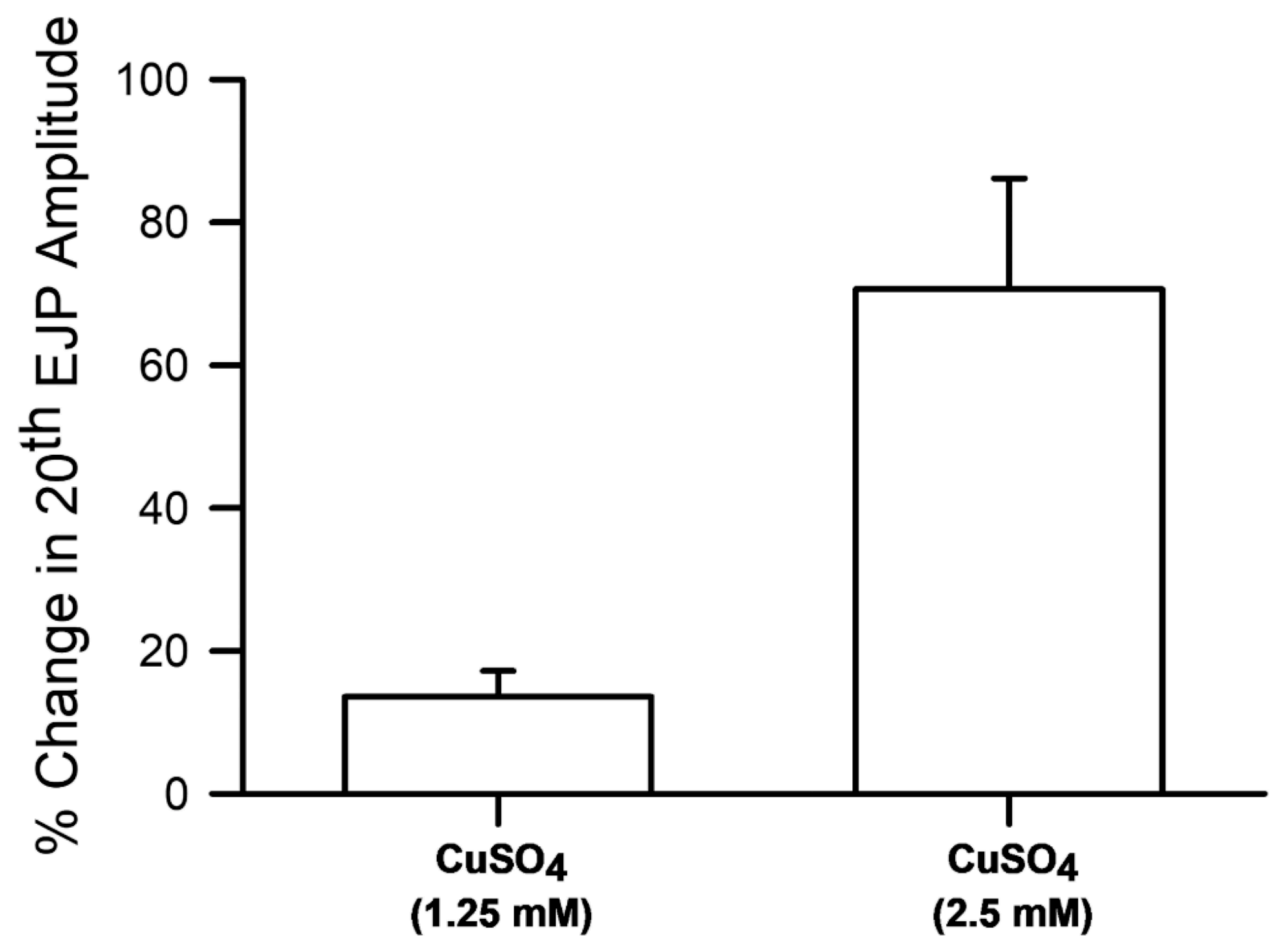
The percentage increase in the amplitude of the 20th excitatory junction potential (EJP) in the train of EJPs delivered at 40 Hz. The average of 50 stimulus responses was determined. The largest averaged response over the exposure time was compared to the averaged amplitude in the initial conditions while exposed to saline alone to determine the percentage change in the amplitude within each preparation. The averaged percentage change of all six preparations was determined (mean +/− SEM). The values of the percentage changes were not normally distributed (Shapiro–Wilk); thus, a Mann–Whitney rank sum test was used, providing *p* = 0.065.

The average of the percentage increase for the six preparations of 1.25 mM CuSO_4_ was not significantly different than the average of the percentage change of the six preparations exposed to 2.5 mM CuSO_4_ (13.9 +/− 3.3; n = 6 vs. 70.9 +/− 15.3; n = 6) (Figure 10; Mann–Whitney Rank Sum Test *p* = 0.065).

### 3.3. Effects of CuSO_4_ on the Resting Membrane Potential

**Figure 11:**
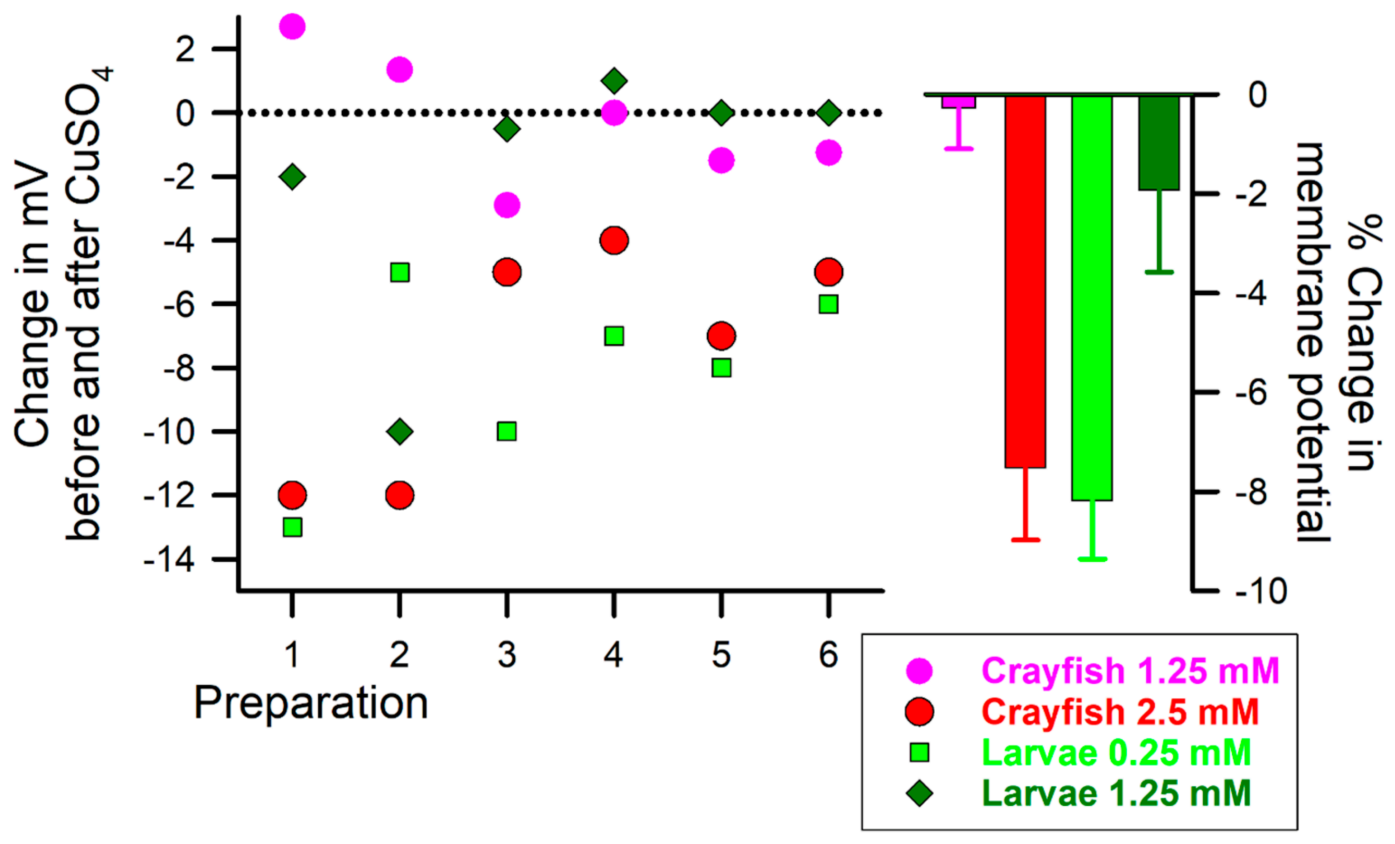
The effect of CuSO_4_ on the resting membrane of the muscle fibers for crayfish and larval muscle fibers. There were no significant effects on resting membrane potential for the crayfish muscle with 1.25 mM CuSO_4_ exposure (paired *t*-test, *p* > 0.05, n = 6) but it was significantly hyperpolarized with exposure to 2.5 mM (paired *t*-test, *p* < 0.05, n = 6). Just the opposite trend was observed for the larval *Drosophila* muscle, with significant hyperpolarization at 0.25 mM (paired *t*-test, *p* < 0.05, n = 6) and no significant difference for 1.25 mM (paired *t*-test, *p* > 0.05, n = 6). The change in the membrane potential was determined as a percentage change within each preparation and averaged (+/−SEM) as shown in the histogram.

The effects of CuSO_4_ on the resting membrane potential for larval *Drosophila* and crayfish muscle were intriguing, as the larval *Drosophila* preparations showed a larger effect at low concentration as compared to a higher concentration of CuSO_4_. However, the crayfish muscle showed smaller changes in the membrane potential for the lower concentration but a larger change for the higher concentrations, as compared to the larval *Drosophila* (Figure 11). There were no significant effects on resting membrane potential for the crayfish muscle with 1.25 mM CuSO_4_ exposure (paired *t*-test, *p* > 0.05, n = 6), but it was significantly hyperpolarized with exposure to 2.5 mM (paired *t*-test, *p* < 0.05, n = 6). Just the opposite trend was observed for the larval *Drosophila* muscle, with a significant hyperpolarization at 0.25 mM (paired *t*-test, *p* < 0.05, n = 6) and no significant difference for 1.25 mM (paired *t*-test, *p* > 0.05, n = 6).

## 4. Discussion

Copper is an essential metal for mammals and other organisms, and is normally consumed in various food sources and through therapeutic supplementation. Inadvertent environmental exposure can also occur in various forms, such as inhalation via industrial and mining activities or by consumption of polluted sources. Since copper is essential for cellular biochemistry, it is naturally detected in most cells but can also accumulate in specific cells when internalized [26,27]. Since the physiological acute effects have not been thoroughly investigated, it is difficult to know how to treat an acute overexposure. Given that as little as 1 g of CuSO_4_ can be toxic to humans when consumed orally [3], and that health food supplements are sold in concentrations of over 500% the daily recommended allowance in one pill, as well as pure CuSO_4_ being readily obtained for home use, such as arts and crafts, on Amazon, more detailed studies regarding its effects on particular tissues are needed. The preliminary investigations undertaken in this study indicate that different research organisms and CuSO_4_ concentrations give unexpectedly varied responses. Such differential responses may also occur among tissue types within mammalian models.

The mechanism to account for its rapid effect in reducing the synaptic efficacy of the larval *Drosophila* NMJ parallels the plausible mechanisms shown for central neuronal communication in rodents in blocking pre-synaptic voltage gated Ca^2+^ channels [40], as well as the ability of Cu^2+^ to act as an antagonist to glutamate receptors (i.e., NMDA and AMPA) [41,42]. The effect of reducing the sensitivity to exogenously applied glutamate in the presence of CuSO_4_ would also be fitting for an antagonist action on the glutamate receptors at the *Drosophila* NMJ, despite not being an NMDA, AMPA or a kainate subtype but rather a quisqualate subtype [43]. The antagonistic action also aids in a possible explanation for the mEJPs becoming smaller in amplitude and disappearing entirely in the background noise.

Shcheglovitov et al. [44] demonstrated that trace metals such as Cu transiently modulate the voltage-dependent gating of mammalian calcium channel Ca(v)2.3, which could potentially affect synaptic transmission and plasticity in the brain. However, this study expressed the particular calcium channels in HEK-293 cells and patch-clamped the channels so that the effects on the whole cell were not addressed. It is yet to be addressed whether Cu has an effect of the Ca_V_ channels at larval *Drosophila* NMJs or crayfish NMJs, but the slight increase in some preparations of the EJP amplitudes in crayfish could possibly be due to similar mechanisms as for the Ca(v)2.3 in lowering the threshold of the channel [44].

However, the hyperpolarization of the larval muscle fibers is an additional phenomenon to be explained. In a study using the olfactory bulb neurons of rats, the voltage-gated K1 channels and input resistance were altered. These changes influenced the repetitive firing of action potentials [40]. It should be noted that the study utilizing the olfactory bulb neurons of rats added CuCl_2_ to the saline to examine the effects of Cu^2+^ [40], while the present study used CuSO_4_. Since the neurons were voltage-clamped, there was no mention of the resting membrane potential being altered in the neurons; however, if input resistance was changed, it may indicate that the leak channels were impacted. The larval *Drosophila* muscles are known to hyperpolarize by influencing K2P channels to open or by overexpressing them [45]. Thus, it is feasible to assume that the low concentration (0.25 mM CuSO_4_) may have stimulated the K2P channels, consistent with evidence that cupric ions selectively bind and modulate K2P channels such as TRAAK [46], as it is unlikely that blocking the Na^+^ leak channels (i.e., NALCN) would account for such large hyperpolarization [45]. Why the higher concentration of CuSO_4_ (1.25 mM) did not produce as much of a hyperpolarization remains to be determined. Since CuCl_2_ was demonstrated to have some ability to block voltage-gated K1 channels [40], there might be some blockage of the K2P channels at higher concentrations. It is now known that Cu can activate a TREK-1 subtype of K2P channels while also blocking the TASK-3 subtype of K2P channels [47]. However, the expression levels and which of the 11 different subtypes of K2P channels in the *Drosophila* genome [48] are expressed in the larval muscle are not yet known, opening the possibility of concentration effects on the subtypes and thus the membrane potential.

One might assume that all the glutamate receptors would be fully desensitized by the continuous exposure to glutamate for the larval *Drosophila* muscle, but a maintained depolarization still occurred with exposure to glutamate. This is likely due to on and off binding on the receptors and possible non-synaptic glutamate receptors on the muscle membrane which were exposed to the bathing media. Therefore, the synaptic glutamate receptors desensitized quickly, reducing the evoked EJPs, while the non-synaptic receptors did not desensitize as rapidly. It was possible that the 3 mM glutamate exposure did not fully desensitize the glutamate receptors, as it was reported that 7 to 10 mM is required [49,50]. However, even with 10 mM glutamate, the muscle maintained depolarization and did not show as rapid or significant desensitization as when co-exposed with CuSO_4_. A possibility is that CuSO_4_ delays glutamate binding or promotes dissociation. However, there are many variables not examined in this study, such as a chemical interaction of glutamate and CuSO_4_ or CuSO_4_ interaction with the ionic salts in the saline or buffer, which may influence the response to glutamate. The focus of this study was demonstrated in the response of glutamate in the presence of CuSO4 in the larval *Drosophila* NMJ, as it was not intended to focus on the mechanisms of glutamate receptor desensitization by glutamate.

The crayfish NMJ revealed something different compared to the *Drosophila* NMJ. CuSO_4_ led to an enhancement in synaptic efficacy by increasing the amplitude of the EJPs, which in part can be attributed to the hyperpolarizing of the postsynaptic muscle. Thus, the driving gradient of the EJP was enhanced by the postsynaptic effect, but it appears that this alone cannot account for the large increase in EJP amplitudes, as a preparation with only 4 mV hyperpolarization of the postsynaptic muscle would produce a 50% increase in the EJP amplitude. It is very likely that there is an effect on the presynaptic membrane of the motor neuron. It is also clear that CuSO_4_ is not blocking the presynaptic voltage-gated Ca^2+^ channels. One possibility is that, like the muscle, the nerve terminal is being hyperpolarized, providing an increased driving gradient for Ca^2+^ when the nerve terminal is depolarized. It is feasible to use calcium indicators to examine such a possibility, but it was beyond the current feasibility of this brief study.

## 5. Conclusions

It is not yet known what length of exposure to CuSO_4_ would lead to detrimental effects on these model synaptic preparations. It is likely that constant exposure in intact larval *Drosophila* or crayfish would prove harmful, as is known for other invertebrates [7]. It was shown that copper had differential survival effects on male and female *Drosophila*, as well as their developmental stage [51]. Such differences in effects may also be present for crayfish and other invertebrates.

There is a need for further studies regarding the detailed mechanisms of action on the blocking of the ionotropic glutamate receptors in the larval *Drosophila*, potentially with outside-out patch studies. This would also help to address the possibility of copper modulating cytoplasmic signaling cascades that subsequently regulate the glutamate receptors for either the larval *Drosophila* or the crayfish preparations [52]. If such a cytoplasmic signaling mechanism were possible, it would need to have rapid action, within seconds, for the larval *Drosophila* preparation. However, for the crayfish preparation, since a slower effect was noted, there is a possibility of a cytoplasmic signaling mechanism increasing the number or type of synaptic glutamate receptors or the kinetics of the receptors as an explanation of a postsynaptic effect. However, the effects may be entirely presynaptic in evoked vesicular fusion events. Additionally, using calcium-sensitive indictors for imaging in both the larval *Drosophila* and crayfish presynaptic motor nerve terminals would allow for a better understanding of whether CuSO_4_ blocks voltage-gated Ca^2+^ channels in the larval *Drosophila* presynaptic terminals and promotes an influx directly or indirectly in the crayfish model.

## Data Availability

The data presented in this study are presented herein and are available from the corresponding author upon specific request.
